# Traumatic Dental Injuries among 12-Year-Old Schoolchildren in the United Arab Emirates

**DOI:** 10.3390/ijerph192013032

**Published:** 2022-10-11

**Authors:** Raghad Hashim, Hebah Alhammadi, Sudhir Varma, Alexander Luke

**Affiliations:** 1 Department of Basic Medical and Dental Sciences, Centre of Medical and Bio-Allied Health Sciences Research, Ajman University, Ajman P.O. Box 346, United Arab Emirates; 2 UAE Ministry of Health, Ajman, United Arab Emirates; 3 Department of Clinical Sciences, Centre of Medical and Bio-Allied Health Sciences Research, Ajman University, Ajman P.O. Box 346, United Arab Emirates

**Keywords:** adolescent trauma, prevalence, risk factors

## Abstract

Aims: This study aimed to evaluate the prevalence of the factors related to traumatic injuries to the permanent incisors of 12-year-old children in the Emirate of Ajman, United Arab Emirates. Methods: A sample of 1008 12-year-old children (510 boys and 498 girls) participated in this study. A multi-stage randomized sampling technique was used to select children from public schools for their inclusion in the sample population. An oral examination was completed by a calibrated examiner after receiving training for traumatic dental injury (TDI) to permanent incisor teeth utilizing a modified version of Ellis’s classification. Data that were related to sociodemographic factors, TDI causes, and where the TDI took place were recorded using a structured questionnaire. Results: Of all of the children that were examined, 9.8% of them had experienced dental trauma. The difference in TDI prevalence between boys (15.9%) and girls (3.9%) was statistically significant (*p* < 0.05). The children whose mothers had low levels of education experienced a higher prevalence of TDI (*p* = 0.001). The children with incisal overjets that were greater than 5 mm or with inadequate lip coverage tended to have experienced dental injuries (*p* < 0.01). The main causes of injury to permanent incisors were collision against an object or person (53.5%) and falling (42.4%). Most of the accidents happened at home (58.6%) and school (25.3%). The most prevalent injuries were enamel fractures (58.7%) and enamel-dentine fractures (34.3%). Conclusions: This research highlighted many predisposing factors for traumatic dental injuries among children. There is an urgent need to initiate detailed public health policies to decrease the prevalence of dental trauma cases, especially in the identified risk group.

## 1. Introduction

Traumatic dental injury (TDI) affects many adolescents, and it considered a challenging public health problem [1,2]. Injuries involving permanent teeth are one of the most common types of trauma to the maxillofacial area [3]. The prevalence of traumatic dental injuries varies greatly, and it ranges from 4% to 58% [4]. Numerous studies have been conducted to identify the probable risk factors that are related to TDI. Male gender [4,5,6,7], incisal overjet more than 5 mm [6,8], inadequate lip coverage [6,8,9], and low socio-economic status [4,10] have been identified as significant risk factors for the incidence of traumatic dental injuries. The evidence suggests that the children with untreated TDIs were 20 times more likely to state that the TDI had an impact on their quality of life (QoL) compared with the children with treated TDIs [11]. It might require hospitalization, and some TDIs lead to lifelong disabilities that negatively influence the children’s development [12]. Approximately 17.7% of the Emirates’ population is made of children and adolescents [13]. Therefore, these injuries among adolescents are of specific concern, as the studies have shown that such injuries are the most prevalent during adolescence [14,15]. There is a shortage of information concerning the epidemiology of dental trauma among the children in the United Arab Emirates (UAE). Understanding some of the factors that are associated with dental trauma will assist in framing strategies to lessen the negative impacts of these injury, especially if we take into consideration that in the UAE, the overall knowledge of mothers [16] and teachers [17] in relation to the emergency management of dental trauma is deficient.

Although the epidemiological studies that are related to TDIs among 12-year-old children have been conducted in many countries, the UAE is without such data. The risk factors that are responsible for TDIs are not crystal clear. The aim of the current study was to determine the prevalence and associated risk factors of the traumatic injury to the permanent anterior teeth among 12-year-old children in the Emirate of Ajman, United Arab Emirates.

## 2. Materials and Methods

Ethical approval for this study was obtained from the ethical committee of Ajman University (AU), College of Dentistry (GD-02-8-1-2018). The total population of Ajman Emirate is 406,537. A pilot study was carried out with 120 12-year-old schoolchildren of both genders that were attending public schools in the Emirate of Ajman. The objective of this was to ensure that the questionnaire that was used was easily understood by the participants. This sample represented 10% of the final sample that included 1221 children. In this cross-sectional study, a multi-stage sampling scheme was implemented; the first stage included all of the public schools in the Emirate of Ajman, and the second stage included all of the 12-year-old children in the designated schools. Half of these schools were selected randomly for their inclusion in this study using a computer program for the generation of random numbers using the lists that were obtained from the Ministry of Education for the Ajman Educational District. An equal probability arrangement was applied by a sampling procedure with the possibility of comparing the school sizes, as the number of children in each school varied. There were 2826 12-year-old children in Ajman. Based on the number of the registered students, eleven schools were randomly and proportionally selected. All of the children that were registered in the selected schools were asked to take part in the survey. A total of 1221 children were selected to take part in this study.

Before the dental examination, a letter was sent to parents of the selected children clarifying the survey’s aim and importance and requesting their child’s inclusion. The informed consent was obtained from the participants’ parents without any bias to the children who chose not to participate. The questionnaire that was attached to the letter requested information regarding the children’s gender and nationality, the parents’ educational level and employment status, and the TDI. The information requested regarding the TDI included the site of the traumatic incident (school, home, “others” such as parks or malls) and details regarding the incident during which the TDI occurred (falls, collisions, traffic accidents, sports, violence, and others).

The children were examined at the school health clinic while sitting on an ordinary chair; daylight and disposable mouth mirrors were used for the dental examinations. Radiographs were not taken, and no treatments were offered. The standard infection control prevention procedures were followed during the examination. The study’s inclusion criteria were a healthy child in a public school with permanent dentition. The exclusion criteria included the presence of deciduous dentition, permanent teeth having been lost due to causes other than trauma, or anodontia affecting the permanent anterior teeth.

To ensure the reliability of the dental examination process, an initial calibration of the examiner (HA) at the College of Dentistry in Ajman University was undertaken, and a replicate dataset was assembled for every tenth participant with a one-day separation between the initial examination and reexamination. The intra- and inter-examiner Kappa scores were 0.85 and 0.80, respectively. The examinations were restricted to the anterior teeth, as other teeth are rarely traumatized. The Ellis classification [18], as it was modified by Holland et al. [19], provided the criteria for the traumatic dental injury in seven classes, as follows: Class I, fracture involving enamel only; Class II, fracture involving enamel and dentine; Class III, fracture involving enamel, dentine, and pulp; Class IV, discoloration as a consequence of tooth concussion, with or without a sinus; Class V, tooth displacement, lateral displacement, intrusion, and extrusion; Class VI, tooth lost as a consequence of trauma; Class VII, tooth restored by a crown or composite subsequent to fracture. Discolored teeth were classified as traumatized when there was an obvious color change with a history of dental trauma. A protrusion that was greater than 5 mm was classified as a case of an increased overjet [19]. An incisor overjet was assessed using a millimeter gauge that was placed between the center of the incisal edges of the mandibular right and maxillary right incisors, while the teeth were in centric occlusion; if these teeth were missing or were mal-positioned, the right central incisors were substituted. The lip competency was documented by observing the children’s faces when they arrived at the examination room, and it was classified as ‘competent’ or ‘incompetent’ based on whether the lips were naturally closed or apart, respectively [20].

A statistical package for the social sciences for Windows, version 20, was used for the analysis. The descriptive statistics included the frequency distribution. The Chi-square test was applied to the relationship between the incidents of the traumatic dental injury and the gender, the parents’ level of education and employment status, the incisal overjet, and an inadequate lip coverage using a significance level of 0.05.

## 3. Results

A total of 1008 12-year-old children (510 boys and 498 girls) took part in this survey. The children who were absent on the day of the dental assessment were not substituted. The response rate was 82.5%. The prevalence of the dental injury within the sample population was 9.8%. There was a significant gender difference (*p* = 0.001) in the prevalence of the traumatic dental injuries, with boys suffering from more TDIs than the girls did. The Emirati children experienced more traumatic dental injuries than the non-Emirati children did, but the difference was not statistically significant. The mothers’ education level was the only socio-demographic variable that showed a statistically significant association with the TDI occurrence, as shown in Table 1.

The children with an incisal overjet that was greater than 5 mm experienced a statistically significant (*p* = 0.001) greater number of dental injuries. Similarly, the children with an inadequate lip coverage experienced more dental injuries compared to those with adequate lip coverage (*p* = 0.001), as shown in Table 2.

The primary cause of trauma was a collision (53.5%), which was followed by falling (42.4%). The least common cause of trauma was by playing sport (1.0%), as shown in Table 3. Most of the accidents occurred at home (58.6%); the remainder of them occurred at school (25.3%) and at “other places” (16.1%), as illustrated in Table 3.

Table 4 presents the classification distribution of the injuries; enamel fracture (58.7%) was the most common injury that was observed, which was followed by an enamel and dentine fracture (34.3%). Only two (2.0%) of 99 injured incisors were treated, either with composite restoration or crowns.

## 4. Discussion

Care should be taken when comparing the TDI prevalence levels due to the variations in the sampling methods, dentition types, and diagnostic criteria that have been used by different researchers. The current study examined 12-year-old children to allow the comparison with previously reported international studies. Certain limitations exist in this study, for example, only teeth-related injuries were recorded since the soft tissue injuries could not be recorded at the time of the clinical examination. Additionally, the study was based on self-reported information, and the responses did not necessarily reflect the actual cause of a TDI. The prevalence of dental injuries (9.8%) that were identified in this study is slightly lower than that which has been reported in many Middle East countries [4,21,22]. Injuries are not random incidents; they are frequently associated with predictable factors. The fact that boys experienced more traumatic dental injuries than girls did agrees with the findings of other researchers [6,23,24]. The fact that girls experienced a low prevalence of dental trauma might be partially explained by there being a higher level of maturity in girls’ behavior compared with the boys, who incline towards risk-taking behaviors [25]. The control imposed on the girls’ behavior by conservative families as a result of Arabs’ cultural and religious perspectives is another possible reason for the low prevalence of TDIs among girls in the United Arab Emirates [26]. In this study, the Emiratis children experienced more traumatic dental injuries than the non-Emiratis did; this might be because the schools that were included in the study were public schools, and the majority of the children that were enrolled in them were Emiratis. Therefore, it is difficult to determine if this reported difference was accurate, as Emirate children were over-presented in the study population. Further studies including children in private schools in the Emirate of Ajman might provide definitive results in the future in this regard.

Few dental trauma studies include socio-economic factors. In this study, only the mothers’ education levels showed a significant relation to the occurrence of traumatic dental injuries, which is in agreement with the findings of Borges et al. [27]. In the literature, the relationship between the socio-economic status and the trauma occurrence is not clear, and there is no agreement among the studies that considered it [28,29]. These inconsistent findings may be attributed in part to the fact that the previous studies were conducted in different countries, which do not share similar socio-economic status patterns. Further research is needed to clarify these relationships.

The current study’s findings indicated that an increased incisor overjet was an important predisposing factor for traumatic dental injury; this is consistent with the findings of recently published systematic reviews [30,31]. Schoolchildren with an inadequate lip coverage had a significantly higher chance of having a TDI, indicating that inappropriate lip coverage is a TDI risk factor [31].

This study clearly illustrates that the main causes of traumatic dental injuries were collision and falling. These findings are in agreement with those of previous reports [4,5]. It is important to take in consideration that a “collision” might present a minor form of violence. In addition, a collision might be a result of play or sport; in both cases, it can be categorized as either a fall or a sports injury. Therefore, it is essential to establish a clear international scheme for recording the cause of dental injuries to facilitate the valid comparisons between countries [32]. The children’s behavior might be directly related to the occurrence of the TDIs; this should always be taken into consideration in emerging strategies to protect dental health. Violent attitudes are a common behavioral risk factor that is associated with a TDI [33]. Most of the traumatic dental injuries happened at home and at school during sports activities, such as swimming, playing soccer, or running; these were the primary activities that were associated directly with the collisions and falls that caused the majority of the children’s traumatic dental injuries. Similar results were reported by other researchers [5,24]. Therefore, special attention should be provided to school and home social education. Providing a supportive social and physical environment in the schools will lead to less traumatic dental injuries occurring [34]. Schoolchildren should be targeted for preventive activities. According to our results, it is essential to consider the strategies for reducing the number of traumatic dental injuries. Children who are involved in contact sports should be encouraged to use mouth guards. It is also essential that children be supervised during physical activities to minimize the chance of falls that lead to traumatic dental injuries. Increasing public awareness is crucial to controlling and minimizing the occurrence of TDIs among children in the United Arab Emirates; public awareness of this issue must be stepped up through national and local campaigns [4].

The majority of TDIs in this study affected the enamel only, and did not necessitate restorative treatment, which is in agreement with other studies [5,35]. Providing a restorative treatment for a traumatized tooth is a daily challenge for dental professionals. One of the critical factors in pediatric restorative dentistry is time because children are frequently distracted and have varying levels of compliance. Although new “bioactive” materials have been introduced recently that overcome these challenges [36], it is important to remember that the success of the restorative treatment should not be connected only to the type of material that is chosen, but it is linked to the modification in lifestyle of the child and the family.

However, nearly one-third of TDIs affected the dentine and 3% of them affected the enamel, dentine, and pulp, which is in agreement with Firmino et al. [37] and de Paiva et al. [38]. This indicated that there were considerable unmet dental treatment needs; therefore, in addition to the preventive strategies highlighting the causal factors of oral conditions, an appropriate post-traumatic dental treatment should be considered to help facilitate clear improvements in oral health and to decrease the number of inequalities. Strategies that are aimed at improving the school environment and increasing public awareness, especially of schoolteachers that are involved in sports activities, might have a positive impact on reducing the number of TDIs. The children’s quality of life might be negatively affected by a traumatic dental injury [39,40]; therefore, it should not be neglected. Further studies are needed to explore the social and personal factors that increase the TDI risk; such data are essential to initiate and implement an effective preventive strategy to reduce the impact of TDIs in the future.

## 5. Conclusions

This study is the first to be conducted in the UAE to address the TDI prevalence and risk factors among adolescents. The findings illustrated that the prevalence of traumatic dental injury to the permanent incisors of 12-year-old children was low. Being male, having a mother with a low level of education, having an overjet more than 5 mm, and having an inadequate lip coverage were significantly related to the prevalence of TDIs among children in the United Arab Emirates.

## Figures and Tables

**Table 1:** Demographic data and distribution of permanent incisor dental injuries in the sample population of 1008 schoolchildren.

	Dental Injury n (%)		No Dental Injury n (%)		All n (%)		*p*-Value for Chi-Square Test
**Gender**							
Boys	81	(15.9)	429	(84.1)	510	(50.6)	0.001
Girls	18	(3.6)	480	(96.4)	498	(49.4)	
**Nationality**							
Emiratis	86	(15.4)	473	(84.6)	559	(55.5)	0.07
Non-Emiratis	23	(5.1)	426	(94.9)	449	(44.5)	
**Mother’s Education**							
Primary	78	(78.0)	22	(22.0)	100	(9.9)	0.001
Secondary	20	(3.5)	544	(96.5)	564	(56.0)	
University	1	(0.3)	343	(99.7)	344	(34.1)	
**Father’s Education**							
Primary	2	(6.5)	29	(93.5)	31	(3.1)	0.134
Secondary	23	(7.7)	277	(92.3)	300	(29.8)	
University	74	(10.9)	603	(89.1)	677	(67.1)	
**Mother’s Employment Status**							
Employed	22	(3.5)	603	(96.5)	625	(62.0)	0.197
Unemployed	77	(20.1)	306	(79.9)	383	(38.0)	
**Father’s Employment Status**							
Employed	94	(9.5)	892	(90.5)	986	(97.8)	0.149
Unemployed	5	(22.7)	17	(77.3)	22	(2.2)	

**Table 2:** Distribution and prevalence of permanent incisor dental injuries by overjet size and lip coverage.

	Dental Injury n (%)		No Dental Injury n (%)		*p*-Value for Chi-Square Test
**Overjet Size**					
≤5 mm	17	(3.1)	529	(96.9)	0.001
>5 mm	82	(17.7)	380	(82.3)	
All	99	(9.8)	909	(90.2)	
**Lip Coverage**					
Adequate	22	(3.7)	569	(96.3)	0.001
Inadequate	77	(18.5)	340	(81.5)	
All	99	(9.8)	909	(90.2)	

**Table 3:** Distribution of causes and places of dental injury among the study population.

	Frequency	(%)
**Cause of Injury**		
Fall	42	(42.4)
Collision	53	(53.5)
Sport	1	(1.0)
Other causes	3	(3.0)
**Place of Dental Injury**		
Home	58	(58.6)
School	25	(25.3)
Other places	16	(16.1)

**Table 4:** Distribution of traumatized teeth according to the type of injury.

	No. of Teeth	(%)
**Type of Injury**		
Enamel fracture alone	58	(58.7)
Enamel & dentine fracture	34	(34.3)
Enamel & dentine fracture with pulp exposure	3	(3.0)
Non-vital tooth with discoloration	0	(0.0)
Displacement	1	(1.0)
Total tooth loss	1	(1.0)
Fracture & restoration	2	(2.0)
**Total**	99	(100)

## Data Availability

The data presented in this study are available on request from the corresponding author.
